# Retroversion and Anteversion of the Uterus

**Published:** 1848

**Authors:** John Evans

**Affiliations:** Professor of Obstetrics, &c., in Rush Medical College


### ARTICLE III.

*Retroversion and Anteversion of the Uterus.* By Jno. Evans, M.D., Professor of Obs., &c., in Rush Medical College.

Retroversion is the displacement next in frequency to prolapsion of the uterus. It may occur at any age of the patient after the organs of generation are developed, but is by far the most common during the early months of pregnancy.

*The Causes* of this dislocation may be divided into the predisposing, and the exciting. Any affection of the system producing debility and relaxation of the tissues, may be regarded as a constitutional predisposing cause, if existing in connection with those local changes in the relation of the parts concerned, termed the local causes. These are, great capacity of the pelvis, enlargements of the fundus of the uterus, elongations, or relaxations of the round and broad ligaments, and distention of the bladder, tumors, &c.

The exciting causes are violent exertions of any kind, as coughing, sneezing, efforts at stool, or in lifting, falls, &c., while the conditions above mentioned are present.

But, perhaps, the most common cause is, exertion while the bladder is distended with urine, and the uterus slightly enlarged by pregnancy. Indeed, it is a matter of surprise, when we observe the very lax attachment of the ligaments, and the loose manner in which the uterus floats in the pelvis, that these conditions should be present, without causing a retroversion in every case, upon violent exertion.

*The Pathology* of retroversion is plain and simple. The uterus is so changed from its natural position, that the fundus is pressed down below the promontory, and occupies the hollow of the sacrum, while the os tincæ is elevated above and rests on the symphysis pubes. By this position, the round and lateral ligaments are extended, and the vagina is thrown forward with the mouth of the uterus, so that in tracing its posterior wall upward, the finger passes over a tumor formed by the uterus, and is brought to the pubis. The rectum is compressed by the fundus, and the neck of the bladder by the mouth of the womb, which, generally, gives rise to difficulties in evacuating urine and fæces, and at the same time, by the irritation of these parts, keeps up a more or less constant desire for it.

The malposition may, in the quiescent condition of the uterus, remain for a considerable length of time without giving rise to intolerable inconvenience, and adhesions are liable to form in cases of long standing, that will prevent reposition, which, in case of pregnancy, must necessarily give rise to abortion, about the fourth month, when the uterus becomes too large to remain in the pelvis, if not earlier.

As in the development of the uterus during the early months of gestation, the enlargement first takes place at the fundus, it is not surprising that we should have the displacement occurring most commonly at this period.

The displacement may occur in connection with diseases of the parts concerned, but, generally, these, unless they relax the attachments of the uterus, load the fundus by enlargements, or, in consequence of tumefaction, push it down, have nothing to do with it. It is, of itself, a purely mechanical derangement.

In those countries where the abdominal viscera are more frequently the seat of disease, as in the south and west, we should expect, both from the congestions and enlargements to which they are liable, and the relaxations produced in the uterine attachments, in common with other parts, that this malposition would be more common. And when to this is added the frequently laborious life of females in the west, necessitating them frequently to make violent exertions at lifting, &c., we may infer that here retroversion will be comparatively of frequent occurrence. But whether these inferences are sustained by facts or not, has not, as yet, been clearly made out by observation.

*The Symptoms* of retroversion of the uterus vary in the different conditions under which it occurs. As a general rule, the larger the uterus at the time, the greater the suffering attending it.

There is pain in the loins and sacral region, passing around the ilium to the pubes, produced by the extension of the ligaments and vagina, and compression of other pelvic viscera—a desire to urinate, with a more or less complete retention of urine, produced by the irritation and compression of the neck of the bladder—often a tenesmus from the irritation and inability to evacuate the bowels, on account of the compression of the rectum—bearing down, resembling labor pains, produced by a sense of the presence of a large tumor, pressing on the perinæum. When it occurs suddenly, and produces much suffering, it is sometimes attended with fainting, paroxyms of hysteria, and frequently by vomiting. Inflammatory fever, with thirst, coated tongue, dry skin, quick pulse, and increased suffering, often supervenes. An examination, per vaginum, will disclose a tumor of greater or less size and density, according to the condition of the uterus, in the cavity of the sacrum, and either an absence of the os tincæ, or it situated against the symphysis pubes, and the parts will be pretty firmly fixed in this position.

*The Diagnosis* of retroversion will be easily made out, as there is no other affection that gives the same relation of the parts, as ascertained by an examination, per vaginum. A tumor in the cavity of the sacrum may resemble it; but the position and mobility of the os tincæ and corpus uteri will enable us to distinguish it from retroversion. The pain, suppression of urine, and sympathetic affections, may attend prolapsus or anteversion; but the os uteri, found by tracing the posterior wall of the vagina, will distinguish retroversion from them or any other possible condition.

*The Terminations* of retroversion are liable to be of the most serious character. The distension of the bladder from the retention of urine, produced by the pressure upon its neck or the urethra, which, perhaps, in some cases, from other causes, existed before the displacement, is liable to result in rupture, and a discharge of urine into the peritoneal cavity, which will be certainly fatal; or in the cellular tissue of the pelvis—scarcely a less dangerous accident, as extensive gangrene must necessarily follow. The large intestine is liable to become impacted in consequence of the pressure on the rectum.

The pressure produced by the uterus against the bladder and rectum, is liable, if long continued, to cause sloughing of these parts, of a most serious character.

An inflammation of the bladder, rectum, uterus, or vagina, and particularly of the ligaments, is liable to result in consequence of the distension and compression to which they are subjected.

In pregnant cases, especially in patients of an irritable system, abortion, with its attendant serious consequences, is liable to ensue; and where the retroversion is old and bound by adhesions, death sometimes results. This is certainly the fate of such a case, if abortion does not occur.

A case of retroversion, probably attended by adhesions, which produced three successive abortions, was reported by Dr. Golliday in the Illinois Med. and Surg. Journal, vol. II, No. 10, in all of which cases the patient did well. In the last case, the patient is stated to have gone to the seventh month of pregnancy, when abortion occurred spontaneously, or from the effects of efforts at reposition.

The adhesions, where the uterus is repeatedly subjected to distensions by pregnancy, might be absorbed, and eventually allow of reposition; and, on this account, we should not be hasty in our interference in such cases.

*The Treatment* of retroversion, as a general rule, is plain and easy, in all cases of recent occurrence. The indications are, to replace the uterus, retain it in its natural position, and meet any irritation or inflammatory symptoms by their appropriate anodyne or antiphlogistic remedies.

The facility with which the first indication can be fulfilled, will depend, to some extent, upon the size of the uterus, the capacity of the pelvis, the amount of inflammation, the condition of the rectum and bladder, the length of time that has elapsed since the displacement occurred, and the formation of adhesions between the uterus and other pelvic viscera, the most liable seat of which is between the fundus and the rectum.

If it be a recent case, there need be no fear of adhesions, and the reposition should be attempted at once. Before proceeding to the fulfillment of this indication, the condition of the patient, in reference to irritation, fever, and inflammation, should be carefully observed. And, in case much evidence of irritation be present, either it should be allayed by the administration of an anodyne, or the chloroform or ether should be given.

If symptoms of inflamation, or if much fever be present, the operation may be safely premised by a free bleeding, in which case, also, on account of the tenderness of the parts to pressure, and the consequent pain produced, will it be more particularly advisable to use an anæsthetic agent. After evacuating the bladder by the use of the catheter, which will sometimes be a little difficult of introduction, on account of the pressure upon the urethra, but by using a male gum-elastic catheter with a stiff stilet, this can be pretty readily overcome, and the catheter can be passed up as high as is necessary, and after procuring a passage from the bowels by an injection, the attempt at reposition may be made.

In some cases this may be effected simply by pressing the fundus through the vagina upward, with one or two fingers, while the patient rests on her knees and breast, to remove the pressure of the abdominal viscera from the uterus, and prevent bearing down.

If this does not succeed readily, I would not pass the finger into the rectum as advised by some, for by it there would be no advantage over the former effort; but I would pass the hand at once into the vagina, and this can scarcely fail of success. The advantages of this procedure are plain; first, by distending the vagina laterally, it is contracted longitudinally, which has a tendency to draw the os tincæ down; second, by the whole hand the uterine tumor can be supported and pressed up by a much broader support than any other means, and hence there is less liability of disturbing the attachments of the ovum and producing abortion; third, with the hand introduced, you can carry the fundus up as far as is necessary for complete reposition.

On the 10th of April, 1847, I was called to see Mrs. ——, with all the symptoms of retroversion. She was near the fourth month of utero gestation, of capacious pelvis, large frame, and strong muscular form—had been in pretty good general health, since she became pregnant, with the exception of one or two slight paroxysms of intermittent fever, within a few days. She had been laboring actively in her kitchen, and after lifting a heavy tub, was thrown into severe pain in the pelvic region, with constant desire to pass urine, without the ability of effecting a discharge.

I attempted a reposition by the fingers, but as the uterus was large, (nearly as large as is possible for it to be in retroversion), the fingers, on pressing the tumor, made too great an indentation to be safely persisted in. I, therefore, after preparing the hand by lubrication with oil, and softening it in warm water, gently passed it into the vagina, in the usual manner and, spreading it out as much as possible to support the uterus, pressed that organ to its proper place.

A slight irritative fever continued for a day or two but subsided after the operation of a cathartic, the bowels became regular, and the voiding of urine easy. On the 16th of September following, I attended her in labor at full term, when she was delivered of a healthy child.

If the retroversion be of long standing, the reposition must be made with much care, lest, if adhesions have formed, lacerations may be produced. Of this, the experienced operator can judge by the amount and manner of resistance offered to reposition. In such cases, attended with pregnancy, the only chance for relief is in abortion, which will often come on spontaneously, or in consequence of efforts at reduction. In some cases it is advised to rupture the membranes, and where the os uteri cannot be reached for this purpose, Dr. Hunter advised to evacuate the liquor amnii by puncturing the uterus through the vagina or rectum, with a small trochar and canula. This has, in several instances, been successfully practiced. Where the uterus is bound down by adhesions, it is better to let it be than run the risk of producing a more serious injury, by attempting to break them up.

After it is replaced, the uterus will generally remain in its proper position, if the causes that produced the displacement be sedulously avoided. Distention of the bladder and active exertion should be especially guarded against.

Where the uterus is growing from the development of an ovum, it will soon be too large for a recurrence of the malposition to be possible. Under other circumstances, the use of a pessary may be servicable, by distending the vagina laterally, and contracting it longitudinally, thus holding the cervex uteri down, and by filling up, to some extent, the space in the cavity of the sacrum, into which the body of the uterus inclines to descend.

When debility attends the displacement, the appropriate tonic treatment for it should be adopted.

### ANTEVERSION.

*Anteversion* is the reverse displacement to retroversion; the difference consisting in the uterus being thrown over in the opposite direction, so as to bring the fundus forward, and the os tincæ backward. It is extremely rare in its occurrence, as might be inferred from the anatomical relation of the parts concerned. The attachment of the bladder to the anterior wall of the vagina, and its elevation above this, between the body of the uterus and the pubes, shows a barrier in the way of the occurrence of this malposition, that will be rarely overcome.

*The Causes* of it are not so clearly defined. Prof. Meigs, of Philadelphia, thinks it depends on contraction of the round ligaments of the uterus. Tumors in the posterior part of the pelvis, and especially of the ovaries, or accumulations of fæces in the rectum and segmoid flexure of the colon, are liable to press the fundus uteri forward, and may thus produce it. It is necessary to this malposition, that the bladder be empty, and it is supposed that a slight enlargement at the fundus uteri favors it. We should, therefore, look for it soon after conception.

Violence or efforts, occurring when the parts are in either of the above conditions, may turn the uterus over forward.

*The Symptoms* of anteversion are, pain, a bearing down sensation, as in retroversion, though not so severe, and a difficulty in retaining urine, sometimes with pain in voiding it, though scarcely ever retention. An examination by the touch alone, will reveal the nature of the case. By this we find a tumor against the anterior wall of the vagina, and the os tincæ carried far back toward or above the promontory of the sacrum.

*The Diagnosis* may readily be made out by a careful examination, per vaginum.

Anteversion has been mistaken for stone in the bladder, in consequence of the fundus pressing a tumor into the cavity of this viscus, which the sound strikes in searching for stone. Leveret mistook the only case of anteversion he saw, for stone, and did not discover his mistake until, after death from lithotomy, it was revealed by dissection.*

The greatest difficulty will be in distinguishing it from tumors in the pelvis; and, as has already been seen, they may both be present, in the relation of cause and effect. The existence of anteversion will be made out by tracing the anterior wall of the vagina, by which we find the body, cervex, and mouth of the uterus in succession, as we pass the finger upward and backward. The posterior wall of the vagina, being traceable forward, will distinguish retroversion.

*Treatment.*—Distension of the bladder is said frequently to relieve anteversion spontaneously, when existing during efforts at stool, &c. The reposition may be effected by pressing the fundus upward, above the pubes, with the fingers, while the patient is in a position to remove the pressure of the abdominal viscera from the pelvis, as recommended in the operation for retroversion.

The after treatment should be the same as for retroversion, except that the bladder may be kept pretty well distended with urine, and thus continually press the fundus back.

* Churchill's Diseases of Females, page 226.

